# Prevalence and Risk Factors of Porcine Cysticercosis in Angónia District, Mozambique

**DOI:** 10.1371/journal.pntd.0000594

**Published:** 2010-02-02

**Authors:** Alberto Pondja, Luís Neves, James Mlangwa, Sónia Afonso, José Fafetine, Arve Lee Willingham, Stig Milan Thamsborg, Maria Vang Johansen

**Affiliations:** 1 Faculty of Veterinary Medicine, Eduardo Mondlane University, Maputo, Mozambique; 2 Faculty of Veterinary Medicine, Sokoine University of Agriculture, Morogoro, Tanzania; 3 Faculty of Life Sciences, University of Copenhagen, Copenhagen, Denmark; Universidad Nacional Autónoma de México, Mexico

## Abstract

*Taenia solium* is an important zoonosis in many developing countries. Cysticercosis poses a serious public health risk and incurs sizeable economic losses to pig production. Because data on the epidemiology of porcine cysticercosis in Mozambique are scarce, the present study was conducted to determine the prevalence and risk factors for porcine cysticercosis. A cross-sectional survey was carried out in 11 villages in Angónia district, Tete province in northwestern Mozambique. Between September and November, 2007, a total of 661 pigs were tested serologically and examined by tongue inspection. Serum samples were tested for the presence of circulating parasite antigen using a monoclonal antibody-based sandwich enzyme-linked immunosorbent assay (Ag-ELISA). In addition, a questionnaire survey to collect information on pig production, occurrence and transmission of porcine cysticercosis, risk factors and awareness of porcine cysticercosis was conducted in the selected households from which pigs were sampled. Two hundred thirty-one samples (34.9%) were found positive by the Ag-ELISA, while by tongue inspection on the same animals cysticerci were detected in 84 pigs (12.7%). Increasing age (OR = 1.63; 95% CI = 1.13–2.37) and free-range pig husbandry system (OR = 3.81; 95% CI = 2.08–7.06) were important risk factors for porcine cysticercosis in the district. The present findings indicate that porcine cysticercosis is endemic in the region, and that increasing pig age and pig husbandry practices contribute significantly to porcine cysticercosis transmission. Further epidemiological studies on the prevalence and transmission of porcine cysticercosis in rural communities in Mozambique are needed to enable collection of more baseline data and implementation of effective control strategies within the country.

## Author Summary

Porcine cysticercosis is an infection of pigs caused by the larval stage of *Taenia solium*, a tapeworm that causes taeniosis in humans. The disease is very common in pigs from many developing countries around the world and poses a serious public health risk and causes significant economic losses in pig production. In Mozambique there is scanty information regarding the occurrence of this disease. Our work conducted in Angónia district, northwestern Mozambique, allowed us to collect important information to evaluate the magnitude and identify the risk factors associated with porcine cysticercosis. This study found that cysticercosis is highly prevalent in pigs and that the free-range husbandry system was by far the main risk factor for the transmission of the disease in the district. These findings should help in the design of control strategies to prevent continuous transmission of this disease in Angónia and in Mozambique.

## Introduction

Porcine cysticercosis is an infection caused by the larval stage of *Taenia solium*, a zoonotic tapeworm that is transmitted among humans and between humans and pigs. The life cycle of this parasite includes pigs as the normal intermediate hosts, harbouring the larval cysticerci, and humans as definitive hosts, harbouring the adult tapeworm causing taeniosis. Humans can also serve as intermediate hosts and develop the cystic form by accidental ingestion of *T. solium* eggs [1],[2]. Human cysticercosis causes a variety of neurological symptoms, most commonly seizures due to cysts in the brain, a condition known as neurocysticercosis [3],[4]. *Taenia solium* cysticercosis is prevalent in humans and pigs in many developing countries of Latin America, Asia, and Africa, where its life cycle is sustained because of the coexistence of poor sanitary conditions, free range management of pigs, and absence or inadequate meat inspection [1],[5],[6]. In Latin America [7], parts of Asia [8] and Africa [9], cysticercosis has been reported as endemic. This disease constitutes a serious but under-recognised public health problem [10] and causes important economic losses due to condemnation of infected pork [4]. In Mozambique, pig production is mainly practiced by smallholders under extensive conditions where food is largely obtained through scavenging. In this system primitive housing is normally provided to protect the animals only during the night. Additionally, there is a lack of slaughterhouse facilities for pigs and inspection and control of pork is poor. These conditions are favourable for the maintenance and spread of *T. solium*. The presence of *T. solium* cysticercosis in Mozambique has been confirmed in a few studies carried out in humans with neurological problems [11]–[14] and in a serological survey of pigs that found a prevalence range of 6.5–33.3% in 11 districts of Tete province using an antibody ELISA test [15]. Additionally, based on old abattoir records, cysticercosis in pigs has been reported from all provinces of the country [16],[17]. However, very few data exist on the epidemiology of porcine cysticercosis in the country. Therefore, the current study was conducted to determine the prevalence and associated risk factors for porcine cysticercosis in Angónia district, Mozambique.

## Material and Methods

### Study area

The survey was conducted in Angónia district located in north-western Mozambique (14°47′S, 34°29′E) and with an altitude that varies from 700 to 1655 meters above sea level. The district was selected following an initial survey conducted by Afonso and others [15], which indicated the presence of free-roaming pigs in the area. Agriculture is the most important economic activity. Pig production, based on an extensive system, dominated by free-ranging pigs, is also an important economic activity in the district.

### Study design and population

A cross-sectional study was conducted between September and November 2007. The sample size to estimate prevalence of porcine cysticercosis, using the formula *n* = *Z^2^PQ/L^2^* [18] and estimating a 30% prevalence, was 322 pigs but to adjust for the multi-stage sampling design used, at least 644 pigs were to be sampled [19]. Households with pigs were identified using the snowballing technique and all pigs in those households were included in the survey. Snowballing is a technique for developing a research sample where existing study subjects recruit future subjects from among their acquaintances. Piglets younger than 2 months, pregnant sows and nursing sows with litters less than 2 months old were excluded from the survey.

### Survey on porcine cysticercosis

All pigs that met our selection criteria were examined for the presence of *T. solium* cysticerci by tongue inspection. Briefly, the pig was firmly restrained in lateral recumbence, a pig snare was used to stabilize the head and a hard wooden stick was used to open the mouth. Using a piece of cotton cloth for grip, the tongue was pulled out, examined and palpated all along its ventral side for the presence of cysticerci. Subsequently, 5 ml of blood were obtained from the cranial vena cava using plain vacutainers. The blood was transported on ice to the laboratory of the Estação Zootécnica de Angónia (EZA) and allowed to clot at 4°C. To obtain serum, the clotted blood was separated by centrifugation, and serum was dispensed into 2 ml labelled aliquots and stored at −20°C until use.

### Household questionnaire

A questionnaire survey to collect information on pig production, occurrence and transmission of *T. solium* cysticercosis, risk factors and awareness of cysticercosis in pigs was carried out in households from the selected villages, in which pigs were sampled as described above. Hygienic and sanitary conditions were inquired about and responses confirmed by direct observation. The respondent in each household was the person taking care of the pigs or the head of the household. It was administered by a field assistant in charge of the agricultural rural extension services in the district using the native language.

### Enzyme-linked immunosorbent assay for detection of circulating antigens

The Ag-ELISA was performed as described by Brandt and others [20] and modified by Dorny and others [21]. Briefly, the serum samples were pre-treated using trichloroacetic acid (TCA) and used in ELISA at a final dilution of 1/4. Two monoclonal antibodies (MoAb) were used in a sandwich ELISA. MoAb B158C11A10 was diluted at 5 µg/ml in carbonate buffer (0.06M/pH 9.6) for coating and a biotinylated MoAb B60H8A4 (1.25 µg/ml in PBS-Tween20 + 1%NBCS) was included as detector antibody. The incubation was carried out at 37°C on a shaker for 30 min for the coating of the first MoAb and for 15 min for all subsequent steps. The substrate solution consisting of ortho phenylenediamine (OPD) and H_2_O_2_ was added and incubated without shaking at 30°C for 15 min. To stop the reaction, 50 µl of H_2_SO_4_ (4N) was added to each well. The plates were read using an ELISA reader at 492 nm. Sera from two known positive pigs (confirmed at slaughter) were used as positive control. To determine the cut-off, the optical density (OD) of each serum sample was compared with a series of 8 reference negative serum samples at a probability level of 0.1% using a modified Student's *t*-test [22].

### Statistical analysis

Data on pig seroprevalence and risk factors were entered and analysed using STATA version 9.1 (Stata Corp., College Station, TX, 2006), and a descriptive analysis was made. A univariate analysis was first performed by calculating odds ratios (OR) for various potential risk factors at the individual level. To investigate whether any presence or lack of association was due to confounding, a multivariate logistic regression analysis was then performed, calculating OR and 95% confidence intervals for risk factors for seropositivity to cysticercosis in pigs, taking into account possible clustering by household.

### Ethical approval

The study protocol was approved by the scientific board at Veterinary Faculty, Eduardo Mondlane University, and the study permissions obtained from the Livestock National Directorate, Mozambique, from village leaders and from the pig owners. Lingual examination and blood sampling on pigs were conducted by a professional veterinary, according to Mozambican guidelines for animal husbandry. Due to high level of illiteracy among villagers, the scientific board at the Veterinary Faculty approved the use of oral consent, and before the commencement of the study it was obtained from pig owners in the presence of a witness, who signed on their behalf.

## Results

### Descriptive

A total of 661 pigs were examined from 306 households in the district of which 383 pigs were from 170 households in Dómuè ward and 278 pigs were from 136 households in Ulóngue ward. In all, 11 villages were visited, 6 in Dómuè and 5 in Ulóngue.

All sampled pigs were of the indigenous breed (black pigs), predominantly females (59%) and about 75% were less than 12 months old. They were mainly left to scavenge during the day and during the dry season and kept in corrals at night and during the rainy season (crop growing season). Relatively few pig farmers (18%) practiced total confinement of their pigs. The pigs were mainly fed maize bran and watermelons, cabbage, and sweet potatoes leaves. Most of the pig farmers (92,5%) kept pigs for both sale and consumption whereas 21.9% of farmers kept pigs for sale and 5.9% for consumption only. A fair proportion of farmers (18.6%) had slaughtered pigs at home, and almost all (99%) did it without inspection. The overall prevalence of porcine cysticercosis was 12.2% (7.8%–23.8%) by tongue examination and 34.9% (22.1%–66.7%) by Ag-ELISA (Table 1).

**Table 1:** Prevalence of porcine cysticercosis in Angónia district, Mozambique based on tongue examination and Ag-ELISA.

Ward	Village	*n*	Positive (%)	Positive (%)
			Tongue Examination	Ag-ELISA
**Dómuè**	Binga	38	6 (15.8)	16 (42.1)
	Campessa	104	13 (12.5)	36 (34.6)
	N'khame	61	7 (11.5)	15 (24.6)
	Seze	62	6 (9.7)	21 (33.9)
	Lilanga	21	5 (23.8)	14 (66.7)
	Ndaula	97	13 (13.4)	29 (29.9)
**Ulóngue**	Ulóngue	130	19 (14.6)	55 (42.3)
	Nhamingona	34	4 (11.8)	14 (41.2)
	Chimuala	11	2 (18.2)	6 (54.5)
	Calómue	77	6 (7.8)	17 (22.1)
	Dzuanga	26	3 (11.5)	8 (30.8)
	Total	661	84 (12.7)	231 (34.9)

Key: *n* = number of pigs.

From a total of 306 households visited, 66% had a female respondent that was taking care of the pigs. Most households (56%) consumed water from wells, and the rest drank water from bore-holes. Few households (5.2%) had no latrines, but most of the latrines (57.9%) in those households with latrines were open and easily accessible for free roaming pigs. Most households (79.1%) reported that had seen cysts in pigs and about 43.5% had sometimes cysticercosis infected pigs. However, few households (17.4%) knew how pigs get the infection and 83% ate pork at least once a month. Of the total number of households visited, 23.5% (11.1%–42.9%) and 56.2% (35.7%–71.4%) had at least one pig positive for porcine cysticercosis by tongue examination and Ag-ELISA, respectively.

### Multiple regression analysis

The factors that were considered in the analysis as risks associated with porcine cysticercosis at animal and household level are presented in Table 2. Considering other factors in the regression model, only age-group and husbandry system were significantly associated with porcine cysticercosis. Age-group of pigs was strongly associated with porcine cysticercosis. While fitted as a linear term to the model, increasing age was significantly associated with porcine cysticercosis. Adult pigs were 63% more likely to be infected with *T. solium* cysticercosis than younger pigs (OR = 1.63; 95% CI = 1.13–2.37). Free-range husbandry system was also significantly associated with porcine cysticercosis compared to confinement system (OR = 3.81; 95% CI = 2.08–7.06).

**Table 2:** Multiple regression analysis for different individual characteristics with odds ratios for infection with porcine cysticercosis in Angónia district, Mozambique.

Risk Factor	Odds Ratio (95% CI)	*p*-Value
**Age group**		
2–4 months	1	
4–7 months	2.77 (1.62–4.75)	<0.001*
8–12 months	3.80 (2.21–6.52)	<0.001*
>12 months	3.56 (2.04–6.19)	<0.001*
**Sex**		
Female	1	
Male	1.19 (0.81–1.77)	0.322
**Pig husbandry system**		
Permanently coralled	1	
Free range	3.81 (2.08–7.06)	<0.001*
**Presence of latrine**		
No	1	
Yes	0.96 (0.35–2.59)	0.929
**Pork consumption**		
No	1	
Yes	0.88 (0.48–1.63)	0.692
**Home slaughter**		
No	1	
Yes	1.58 (0.87–2.89)	0.135
**Observation of cysts in pork**		
No	1	
Yes	1.28 (0.72–2.27)	0.404
**Knowledge of how pigs get infected**		
Yes	1	
No	1.03 (0.52–2.06)	0.928

Key: * statistically significant at 95% CI.

## Discussion

This study has investigated the prevalence and the potential risk factors associated to *T. solium* cysticercosis in pigs in Angónia district. The overall prevalence based on detection of circulating antigens (34.9%) in this study indicates that porcine cysticercosis is highly prevalent in the district. Similar results were reported by Afonso and others [15] in a study conducted in Tete province using an antibody detection (Ab-ELISA) test in animals. This high value suggests that pigs in Angónia district are exposed to *T. solium* eggs. Most households (94.8%) had latrines but yet had infected pigs. However, the prevalence of porcine cysticercosis did not differ between households with and without latrines. This agrees with a study conducted in Cameroon [23], which found no statistical difference in cysticercosis prevalence in pigs raised in households with or without latrines. Similarly, a study in Mexico [24] found no association between the absence of latrines and the prevalence of porcine cysticercosis. Impressively, indoor latrines have been shown in a Mexican study to be positively associated with porcine cysticercosis in a setting where the outlets of the latrines were deliberately put in the pig pens [24]. In contrast surveys conducted in Tanzania [25] and Zambia [26] showed that the prevalence of porcine cysticercosis was considerably higher in pigs reared in households lacking latrines than in those reared in households that had latrines. This finding could suggest that, either farmers in Angónia district were not using the latrines, or pigs had access to the latrines since most of them were open and easily accessible for roaming pigs. Additionally, farmers spent most of their time in the fields, and might have been practicing indiscriminate defecation.

The lack of association between latrines and porcine cysticercosis might have been due to the fact that pigs were allowed to roam freely in both households with and without latrines. It has been shown in this study that pigs from households that practiced free-range system were more likely to be positive for cysticercosis than corralled pigs. Therefore, free range pig management system represented by far the most important risk factor for porcine cysticercosis in Angónia. Free ranging has been previously associated with porcine cysticercosis in Mexico [24] and Africa [23],[25],[26], as it allowed pigs easy access to human faeces.

The present study demonstrated that the prevalence of porcine cysticercosis increased with age of the pigs. These results are in agreement with those reported in Mexico [24], Cameroon [23] and Peru [27]. This may indicate either that older animals might have had longer exposure than the younger ones or that younger pigs are protected through the initial exposure period, perhaps via maternal transfer of antibodies, but become susceptible later. Maternal antibodies are protective for other larval cestode infections [28] and have been shown to slowly decrease in piglets born to cysticercosis infected sows [29]. However, other studies did not report positive association [25],[30].

Although many households (79.1%) reported that have observed cysts in pigs, few (17.4%) were aware of how pigs got the infection. However, the prevalence of porcine cysticercosis among households with knowledge of the causative agent of cysticercosis was similar to that without such knowledge. According to Sarti and others [24] the lack of knowledge about the parasite life-cycle and the way socio-economic conditions (sanitation, pig husbandry, contact of pigs with human faeces) affect transmission helps to promote transmission of *T. solium* in rural communities. On the other hand, although education campaigns studies have demonstrated that villagers understand the role of *T. solium* infections in pigs and humans, this knowledge does not appear to result in dramatic changes in risk behaviour [2]. However, health education campaigns have been effective in reducing the transmission rates of *T. solium* infections in humans and pigs [31]. Recently, a study conducted in Tanzania demonstrated that health education intervention led to an important reduction in the incidence rate of porcine cysticercosis and reported cases of household consumption of infected pork, despite minimal improvement in behaviour and practices related to its transmission [32].

Our findings give clear evidence that porcine cysticercosis is endemic in Angónia district, and that increasing age and free ranging of the pigs increase the likelihood of exposure to *T. solium* eggs and thereby the transmission of the disease within the study community. Given the importance of this disease, educational programmes should be initiated to build awareness on the transmission of *T. solium* infections, and further epidemiological studies in rural communities in Mozambique are required to allow collection of more baseline data and effective implementation of control strategies.

## Supporting Information

Checklist S1 STROBE checklist.
(0.09 MB DOC)
