# Supplementary material for: Prevalence and Risk Factors of Porcine Cysticercosis in Angónia District, Mozambique
Source: PLoS Negl Trop Dis. 2010 Feb 2;4(2):e594. doi: 10.1371/journal.pntd.0000594 (PMC2814857; doi:10.1371/journal.pntd.0000594)
Supplement: Checklist S1 — STROBE checklist. (0.09 MB DOC) [file pntd.0000594.s001.doc]

STROBE Statement

|  | Item No |  |
| --- | --- | --- |
| **Title and abstract** | 1 | Prevalence and risk factors of porcine cysticercosis in Angónia district, Mozambique |
|  |  | To determine the prevalence and risk factors of porcine cysticercosis, a cross-sectional survey was carried out in 11 villages in Angónia district, Tete province in north-western Mozambique. Between September and November, 2007, a total of 661 pigs were tested serologically and examined by tongue inspection. Serum samples were tested for the presence of circulating parasite antigen using a monoclonal antibody-based sandwich enzyme-linked immunosorbent assay (Ag-ELISA). Two hundred thirty one samples (34.9%) were found positive by the Ag-ELISA, while by tongue inspection on the same animals cysticerci were detected in 84 pigs (12.7%). Increasing age (OR = 1.63; 95% CI = 1.13 – 2.37) and free-range pig husbandry system (OR = 3.81; 95% CI = 2.08 – 7.06) were important risk factors for porcine cysticercosis in the district. This study has identified some community practices that should be addressed in community-led control programmes to prevent continuous transmission of *T. solium* cysticercosis. |
| Introduction | | |
| Background/rationale | 2 | Porcine cysticercosis is an infection of pigs caused by the larval stage of *Taenia solium*, a zoonotic tapeworm that is transmitted among humans and between humans and pigs. *Taenia solium* cysticercosis is prevalent in humans and pigs in many developing countries of Latin America, Asia, and Africa, where its life cycle is sustained because of the coexistence of poor sanitary conditions, free ranging of pigs, and absence or inadequate meat inspection (Gonzalez et al., 2001). This disease constitutes a serious but under-recognised public health problem and causes important economic losses due to condemnation of infected pork (Pawlowski et al., 2005). In Mozambique, *T. solium* cysticercosis in Mozambique has been confirmed from few studies carried out in humans with neurological problems (Noormahomed et al., 2003) and from a serological survey of pigs that found a prevalence range of 6.5-33.3% (Afonso et al., 2001). Additionally, based on abattoir records, cysticercosis in pigs has been reported from all provinces of the country. However, very few data exist on the epidemiology of porcine cysticercosis in the country. Therefore, the current study was conducted to determine the prevalence and associated risk factors for porcine cysticercosis in Angónia district, Mozambique. |
| Objectives | 3 | Determine the prevalence of porcine cysticercosis in Angónia district  Determine the risk factors associated to porcine cysticercosis in Angónia district |
| Methods | | |
| Study design | 4 | Cross-sectional study |
| Setting | 5 | Angónia district is located in north-western Mozambique (14º47´S, 34º29´E), with an altitude that varies from 700 to 1655 meters above sea level. Agriculture is the most important economic activity. Pig production, based on an extensive system, dominated by free-ranging pigs, is also an important economic activity in the district. Preliminary visit to approach and explain the objective of the study was made in April 2007. Data collection was conducted between September and November 2007. |
| Participants | 6 | Households with pigs were identified using the snowballing technique and all pigs in those households were included in the survey. Piglets younger than 2 months, pregnant sows and nursing sows with litters less than 2 months old were excluded from the survey |
|  |  |  |
| Variables | 7 | Outcome variable: Prevalence of porcine cysticercosis  Factors: Age of pigs, Sex, Pig husbandry system, Presence of latrine, Pork consumption, Home slaughter, Observation of cysts in pork, Knowledge of how pigs get infected. |
| Data sources/ measurement | 8* | Prevalence: Positive cases of porcine cysticercosis were assessed through lingual examination and serum testing using Ag-ELISA  Age of pigs was inquired and corroborated with teeth eruption  Pig husbandry systems, presence of latrine, pork consumption, and home slaughter were inquired (questionnaire) and complemented by direct observation, when applicable.  Observation of cysts in pork and knowledge of how pigs get infected were inquired. |
| Bias | 9 | Random selection of 11 villages from 16 district villages. |
| Study size | 10 | The sample size to estimate prevalence of porcine cysticercosis was calculated according to the formula: *n = Z^2^PQ/L^2^*. An estimated prevalence of 30 %, based on former studies was used to obtain a sample size of 322 pigs. This number was doubled to adjust for the multi-stage sampling design used, thus, at least 644 pigs were to be sampled. |
| Quantitative variables | 11 | Age was handled either as a linear term or grouping in the analysis. |
| Statistical methods | 12 | A univariate analysis was first performed by calculating odds ratios for various potential risk factors at the individual level. To investigate whether any presence or lack of association was due to confounding, a multivariate logistic regression analysis was then performed, calculating odds ratios and 95% confidence intervals for risk factors for porcine seropositivity to cysticercosis, taking into account possible clustering by household. |

Continued on next page

| Results | | |
| --- | --- | --- |
| Participants | 13* |  |
| Descriptive data | 14* | A total of 661 pigs were examined from 306 households in the district of which 383 pigs were from 170 households in Dómuè ward and 278 pigs were from 136 households in Ulóngue ward. In all, 11 villages were visited, 6 in Dómuè and 5 in Ulóngue. All sampled pigs were of the indigenous breed (black pigs), predominantly females (59%) and about 75% were less than 12 months old. They were mainly left to scavenge during the day and during the dry season and kept in corrals at night and during the rainy season (crop growing season). Relatively few pig farmers (18%) practiced total confinement of their pigs. The pigs were mainly fed maize bran and watermelons, cabbage, and sweet potatoes leaves. Most of the pig farmers (92,5%) kept pigs for both sale and consumption whereas 21.9% of farmers kept pigs for sale and 5.9% for consumption only. A fair proportion of farmers (18.6%) had slaughtered pigs at home, and almost all (99%) did it without inspection.  From a total of 306 households visited, 66% had a female respondent that was taking care of the pigs. Most households (56%) consumed water from wells, and the rest drank water from bore-holes. Few households (5.2%) had no latrines, but most of the latrines (57.9%) in those households with latrines were open and easily accessible for free roaming pigs. Most households (79.1%) reported that had seen cysts in pigs and about 43.5% had sometimes cysticercosis infected pigs. However, few households (17.4%) knew how pigs get the infection and 83% ate pork at least once a month. |
| Outcome data | 15* | Two hundred thirty one samples (34.9%) were found positive by the Ag-ELISA, while by tongue inspection on the same animals cysticerci were detected in 84 pigs (12.7%). |
| Main results | 16 | The overall prevalence of porcine cysticercosis was 12.2% (7.8%-23.8%) by tongue examination and 34.9% (22.1%- 66.7%) by Ag-ELISA. The overall prevalence in this study indicates that porcine cysticercosis is highly prevalent in the district.  The factors that were significantly associated with porcine cysticercosis were age and pig husbandry system. Adult pigs were 63% more likely to be infected with *T. solium* cysticercosis than younger pigs (OR = 1.63; 95% CI = 1.13 – 2.37). Free range husbandry system was also significantly associated with porcine cysticercosis compared to corralling system (OR = 3.81; 95% CI = 2.08 – 7.06). |
| Other analyses | 17 |  |
| Discussion | | |
| Key results | 18 | The overall prevalence in this study indicates that porcine cysticercosis is highly prevalent in the district. Similar results were reported by Afonso and others (2001) in a study conducted in Tete province using an antibody detection (Ab-ELISA) test in animals. This high value suggests that pigs in Angónia district are exposed to *T. solium* eggs. Most households (94.8%) had latrines but yet had infected pigs. However, the prevalence of porcine cysticercosis did not differ between households with and without latrines. This finding could suggest that, either farmers in Angónia district were not using the latrines, or pigs had access to the latrines since most of them were open and easily accessible for roaming pigs. Additionally, farmers spent most of their time in the fields, and might have been practicing indiscriminate defecation.  The lack of association between latrines and porcine cysticercosis might have been due to the fact that pigs were allowed to roam freely in both households with and without latrines. It has been shown in this study that pigs from households that practiced free-range system were more likely to be positive for cysticercosis than corralled pigs. Therefore, free range pig management system represented by far the most important risk factor for porcine cysticercosis in Angónia. Free ranging has been previously associated with porcine cysticercosis in Mexico and Africa, as it allowed pigs easy access to human faeces.  The present study demonstrated that the prevalence of porcine cysticercosis increased with age of the pigs. This may indicate either that older animals might have had longer exposure than the younger ones or that younger pigs are protected through the initial exposure period, perhaps via maternal transfer of antibodies, but become susceptible later. Maternal antibodies are protective for other larval cestode infections and have been shown to slowly decrease in piglets born to cysticercosis infected sows. |
| Limitations | 19 | The sampling method, snowball sampling, whereby initial sampling frames are constructed by “informants” who provide researchers with referrals to other units meeting certain criteria. Snowball sampling is often considered as a biased from of sampling because selection is non-random, since in the absence of knowledge of individual inclusion probabilities in different waves of the snowball sample, unbiased estimation is not possible. However, in the present study, because there was no information regarding pig production/population in the study area, this method was used and we sampled to redundancy to assure that all pig farmers were included in a randomly selected village, and therefore minimize the doubt on the representativeness since using this procedure, the sampling frame is often biased towards the inclusion of units with dense networks of interrelationships. |
| Interpretation | 20 | The overall prevalence in this study indicates that porcine cysticercosis is highly prevalent in the district, and is associated to free-range pig husbandry and increasing age of pigs. These results are similar with others reported in the country and elsewhere, under similar settings. This study has identified, like others, some community practices that should be addressed in community-led control programmes to prevent continuous transmission of *T. solium* cysticercosis in endemic areas. |
| Generalisability | 21 | The results of this study can be generalized to the settings of Mozambique where pig production, alike in Angónia district, is mainly developed by poor smallholder farmers based in rural communities, where free-range is the most common form of pig husbandry, sanitation and hygiene is poor, and there is a lack of knowledge regarding *T. solium* infections. |
| Other information | | |
| Funding | 22 | This study was conducted under the CESA-project (Cross-disciplinary risk assessment of Cysticercosis in Eastern and Southern Africa), funded by the Danish International Development Agency (DANIDA), file no. 104.Dan.8.L.721. |

*Give information separately for cases and controls in case-control studies and, if applicable, for exposed and unexposed groups in cohort and cross-sectional studies.
